# Does EMG control lead to distinct motor adaptation?

**DOI:** 10.3389/fnins.2014.00302

**Published:** 2014-09-30

**Authors:** Reva E. Johnson, Konrad P. Kording, Levi J. Hargrove, Jonathon W. Sensinger

**Affiliations:** ^1^Center for Bionic Medicine, Rehabilitation Institute of ChicagoChicago, IL, USA; ^2^Department of Biomedical Engineering, Northwestern UniversityEvanston, IL, USA; ^3^Department of Physical Medicine and Rehabilitation, Northwestern University and Rehabilitation Institute of ChicagoChicago, IL, USA; ^4^Department of Engineering Sciences and Applied Mathematics, Northwestern UniversityEvanston, IL, USA; ^5^Department of Physiology, Northwestern UniversityChicago, IL, USA; ^6^Institute of Biomedical Engineering, University of New BrunswickFredericton, NB, Canada; ^7^Department of Electrical and Computer Engineering, University of New BrunswickFredericton, NB, Canada

**Keywords:** prosthesis control, EMG, motor adaptation, uncertainty, sensory feedback

## Abstract

Powered prostheses are controlled using electromyographic (EMG) signals, which may introduce high levels of uncertainty even for simple tasks. According to Bayesian theories, higher uncertainty should influence how the brain adapts motor commands in response to perceived errors. Such adaptation may critically influence how patients interact with their prosthetic devices; however, we do not yet understand adaptation behavior with EMG control. Models of adaptation can offer insights on movement planning and feedback correction, but we first need to establish their validity for EMG control interfaces. Here we created a simplified comparison of prosthesis and able-bodied control by studying adaptation with three control interfaces: joint angle, joint torque, and EMG. Subjects used each of the control interfaces to perform a target-directed task with random visual perturbations. We investigated how control interface and visual uncertainty affected trial-by-trial adaptation. As predicted by Bayesian models, increased errors and decreased visual uncertainty led to faster adaptation. The control interface had no significant effect beyond influencing error sizes. This result suggests that Bayesian models are useful for describing prosthesis control and could facilitate further investigation to characterize the uncertainty faced by prosthesis users. A better understanding of factors affecting movement uncertainty will guide sensory feedback strategies for powered prostheses and clarify what feedback information best improves control.

## Introduction

Powered upper limb prostheses offer the possibility of restoring abilities lost due to amputation; however, lack of kinesthetic feedback requires users to devote constant visual attention to every task. Myoelectric prostheses are controlled using electromyographic (EMG) signals, which are highly variable byproducts of muscle contraction (Clancy et al., 2002). Despite recent improvements in prosthesis technology (Weir and Sensinger, 2009) and EMG signal processing (Parker et al., 2006), prosthesis movements are imprecise, and many amputees abandon their devices out of frustration (Biddiss and Chau, 2007; Biddiss et al., 2007). Providing additional sensory feedback is an intuitive solution, but this has not yet been implemented clinically (Antfolk et al., 2013). To provide effective sensory feedback, we need to understand how amputees incorporate feedback information into movement planning.

The role of feedback in able-bodied movement is described well by a sensorimotor adaptation framework. This framework theorizes that the nervous system coordinates movements by predicting the state of the body and correcting this prediction using sensory feedback (Wolpert et al., 1995). The state prediction and feedback processes are each estimated with some uncertainty, caused by many possible factors (Orbán and Wolpert, 2011). The relative uncertainties of state prediction and sensory feedback determine how these two sources of information are combined (Kording and Wolpert, 2004). For example, if sensory feedback is very uncertain (due to increased sensory variability, e.g., blurred vision) the brain will rely more heavily on the feedforward state prediction. Thus, the impact of sensory feedback depends on the uncertainty of both the sensory and motor information.

Uncertainty levels are presumably high during prosthesis use, due to EMG signal variability and limited sensory feedback. Some studies suggest that adding sensory feedback reduces uncertainty (Wheeler et al., 2010; Saunders and Vijayakumar, 2011), although others report either no improvement or conflicting results (Antfolk et al., 2013). In many cases, the reasons for the ineffectiveness of sensory feedback remain unclear: Are users perceiving high uncertainty in the feedback? Are users relying entirely on feedforward state predictions, and ignoring feedback? Are users able to generate state predictions at all when using EMG control? To accurately describe prosthesis control and implement effective sensory feedback, we must determine the effects of high motor uncertainty and control signal modality on adaptation.

Several possible factors may affect adaptation with EMG control. High motor variability may affect adaptation rate (Burge et al., 2008) and estimation of error relevance (Wei and Kording, 2009). The lack of direct sensory feedback from EMG activity may increase feedback uncertainty. Central nervous system processes [e.g., efference copy formation (Poulet and Hedwig, 2007) and internal modeling of system dynamics (Kawato, 1999)] are not well-understood for EMG control, which relies on indirect biological signals. This study considers the effect of high motor variability by measuring the effect of mean error on adaptation rate; other factors are considered collectively by measuring the effect of control interface on adaptation rate.

We investigated trial-by-trial adaptation with two levels of feedback uncertainty and three different control interfaces: joint angle, joint torque, and EMG. The control interface influenced the motor uncertainty of the user and enabled a simplified comparison of adaptation behavior between prosthesis and able-bodied control. Trial-by-trial adaptation rate was examined as a function of feedback uncertainty, control interface, and mean error.

## Methods

Eight able-bodied subjects (three female, five male) participated in this experiment, which was approved by the Northwestern University Institutional Review Board. Subjects were between 23 and 32 years old.

### Experimental protocol

**Figure 1:**
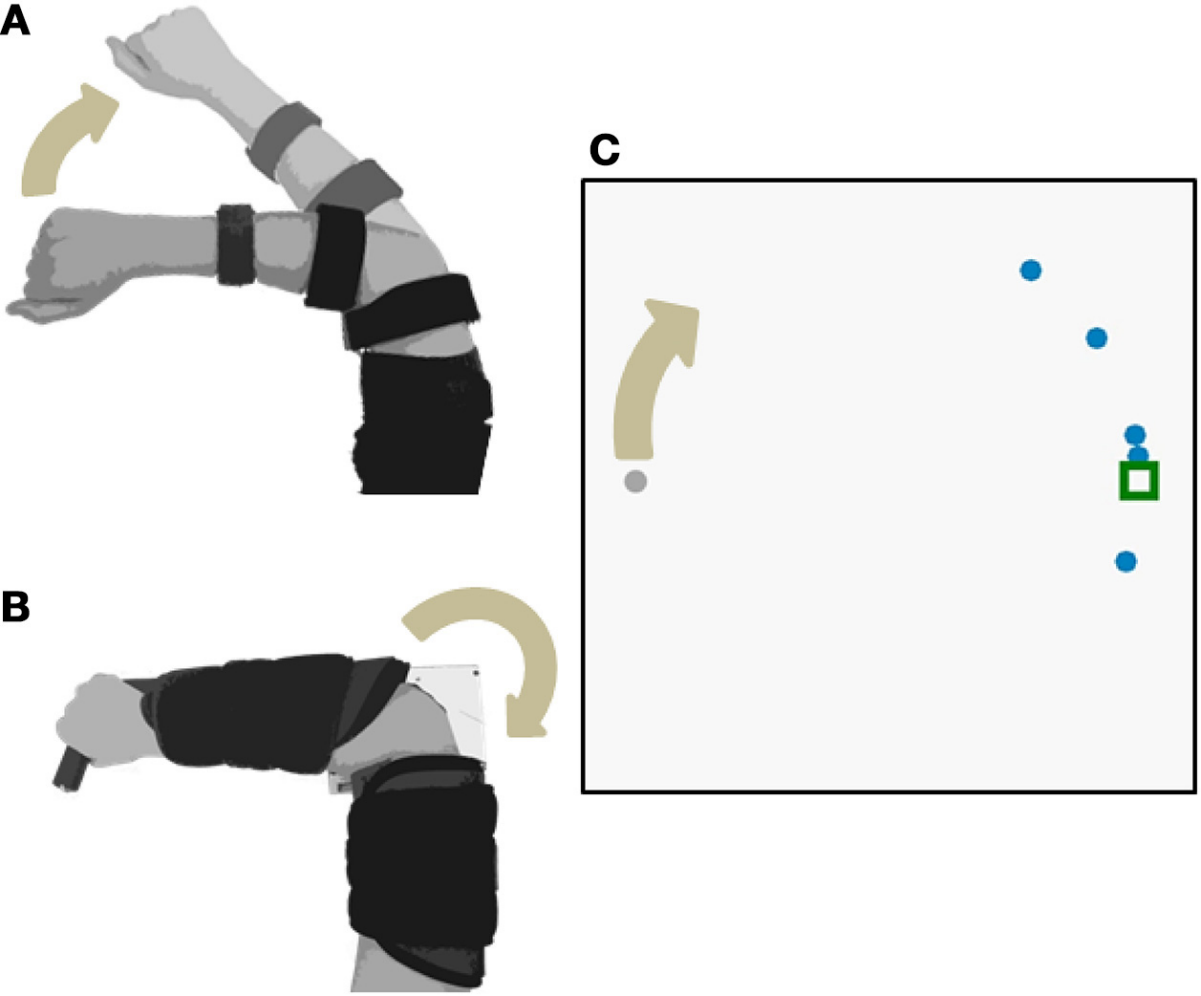
**Experimental Setup**. Subjects used elbow extension to move a cursor with three different control interfaces: **(A)** joint angle and **(B)** joint torque and EMG. The joint angle control interface used isotonic contractions; joint torque and EMG control interfaces both used isometric contractions. The cursor moved along a 1-DOF circular track **(C)**.

Subjects sat comfortably in front of a computer display screen (shown in Figure 1C). They used elbow extension movements to control a virtual cursor along a single degree-of-freedom (DOF) circular track (radius = 13 cm). The cursor started at the left side of the circle (180°) and a target remained stationary at the right side of the circle (0°). The start of each trial was indicated by an audio signal triggered by the experimenter. Subjects had 3 s to move the cursor from the starting position to the target. The cursor then returned to the starting position.

Each experiment comprised three phases: familiarization, training, and testing. The familiarization phase consisted of 10 trials, in which the cursor was displayed as one dot that was unperturbed and visible throughout the trial. In the training phase, the cursor was still unperturbed and displayed as one dot, but visual feedback was taken away 0.5 s into the trial. The cursor reappeared after the trial to give 100 ms of terminal feedback (Baddeley et al., 2003; similar to Wei and Kording, 2010; and others). Training continued until the subject was able to complete 10 trials with an average error of under 20° (this usually required 15–20 trials). In the testing phase, subjects were given only terminal visual feedback. The testing phase included 4 blocks of 75 trials each, with approximately 2 min of rest between blocks.

During the testing phase, visual perturbations were applied to the displayed cursor endpoint. Perturbations were randomly distributed between −40°, 0°, and 40°. Subjects were encouraged to hit the target as accurately as possible, and were instructed that the terminal visual feedback represented the true cursor position.

Two levels of feedback uncertainty were created in the testing phase by displaying the final cursor position as either one or five dots (an approach used previously by Tassinari et al., 2006; Wei and Kording, 2010; and others). When subjects saw the cursor as one dot, feedback uncertainty was low. When subjects saw five dots, feedback uncertainty was high. The location of the five dots was drawn from a Gaussian distribution with the mean as the cursor position and a standard deviation of 40°. Level of feedback uncertainty was randomly assigned for each trial.

### Control interfaces

Subjects completed the experimental protocol once for each of the control interfaces: joint angle, joint torque, and EMG. Each control interface was tested on separate days, in randomized order. The experimental setups for each control interface are shown in Figure 1.

#### Joint angle control interface

In the joint angle control interface, the subject extended the right elbow (isotonic contraction). An electrogoniometer (Biometrics Ltd) measured the elbow angle of the right arm (Figure 1A). The end blocks of the goniometer were attached to a hinged two-bar planar linkage. One link was fixed to a flat surface and strapped to the subject's upper arm. The other link was free to rotate, slid easily across the flat surface, and was strapped to the subject's lower arm. A mechanical stop prevented the subject from flexing past 45° and served as the starting position for each trial. The subject's view of the arm was blocked. The angle output of the goniometer was filtered with a low-pass cutoff frequency of 50 Hz. Elbow flexion of 45–135° was mapped to 0–360° of the circular cursor track.

#### Joint torque and EMG control interfaces

In the torque and EMG control interfaces, the subject generated isometric extension torque about the elbow (Figure 1B). Elbow extension torque was measured by a reaction torque sensor (TFF40, Futek Inc.). EMG activity during isometric elbow extension was measured by a self-adhesive bipolar electrode (Bagnoli™, Delsys Inc.) placed over the lateral head of the triceps brachii. The subject's right arm was strapped into a modified elbow brace that restricted motion (Elbow RANGER Motion Control, ProCare®). The lower arm portion of the brace was fixed to a horizontal link that coupled to the shaft of the torque sensor. The upper arm portion of the brace was fixed to the housing of the torque sensor.

The control signals were calibrated such that equal effort was required to move the cursor for both torque and EMG control interfaces. Subjects exerted approximately 4 N-m of extension torque for 10 s by viewing a screen that indicated their current torque and the goal torque. Both torque and EMG control signals were normalized to the mean absolute values recorded during the 10 s calibration. Control signals were high-pass filtered at 0.1 Hz, rectified, low-pass filtered at 5 Hz, normalized, and mapped to cursor angle with the following transfer function:

$$\frac{\theta \left(s\right)}{u\left(s\right)}=\frac{1250}{s^{2}+11s}.$$

Similar dynamics are commonly used as an EMG filter for clinical prostheses (Sensinger and Weir, 2008). Parameters were chosen to match the dynamics of a typical prosthetic arm—the LTI Boston Digital™ elbow (Heckathorne, 2004).

## Results

We investigated the influence of control interface on trial-by-trial adaption to visual perturbations with two levels of feedback uncertainty. Subjects used three control interfaces—elbow extension angle, torque, and EMG—to move a cursor toward a stationary target. Terminal visual feedback was displayed as one dot (low feedback uncertainty) or five dots (high feedback uncertainty). Adaptation rate was assessed as a function of control interface, feedback uncertainty level, and mean absolute endpoint error.

**Figure 2:**
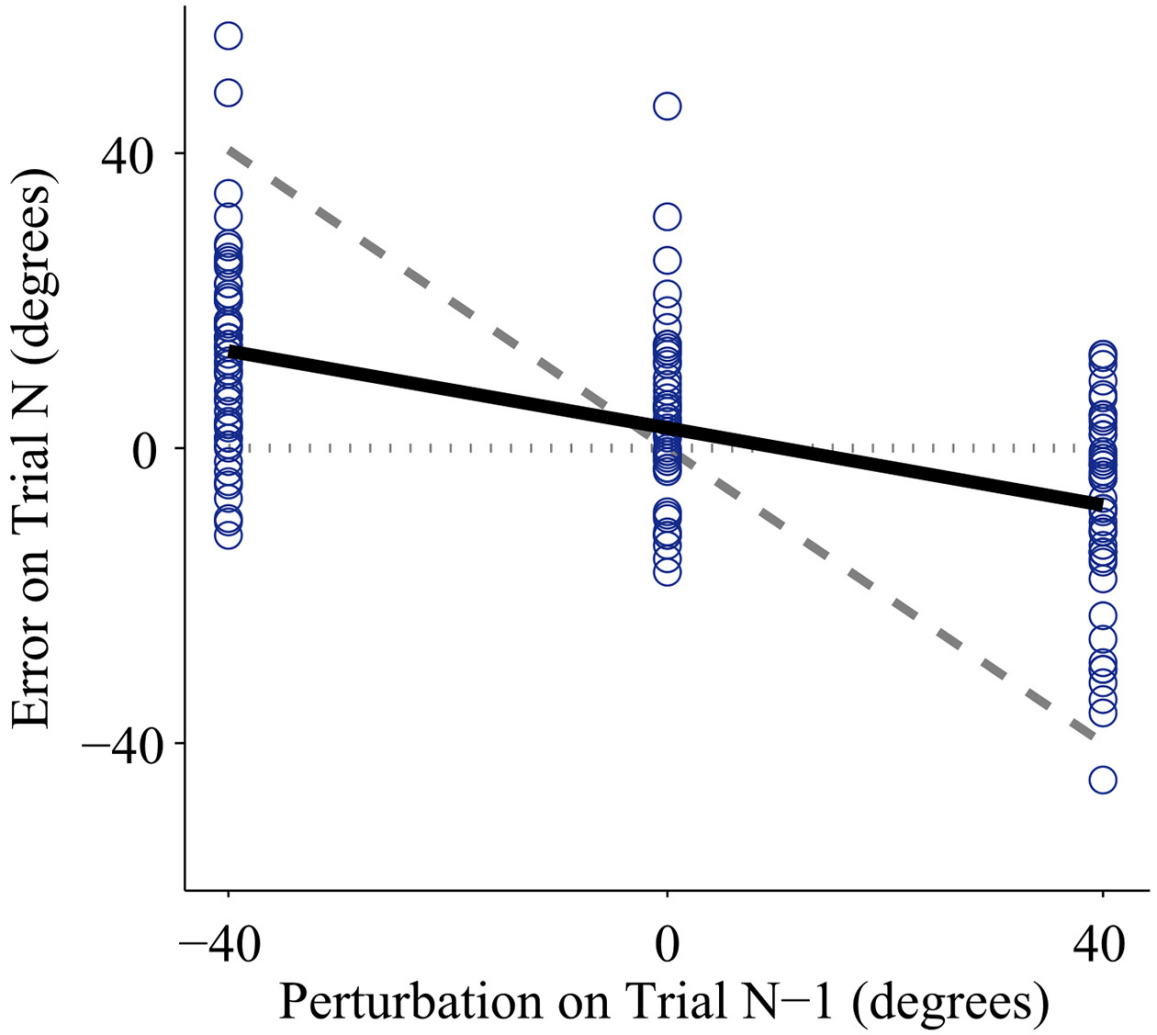
**Representative data from one subject using the joint angle control interface with low feedback uncertainty**. Individual trials are plotted as circles. The x-axis shows the perturbation size for a trial with one-dot terminal feedback, and the y-axis shows the error on the following trial (error is defined as the unperturbed or true distance between the cursor and the target). Adaptation rate is defined as the slope of the linear regression between the unperturbed error of trial (N) and the perturbation of trial (N-1). The regression is plotted as the bold solid line. If a subject showed no adaptation, the regression slope would equal zero, illustrated by the horizontal dotted line. If a subject showed complete adaptation, the regression slope would equal −1, illustrated by the dashed line. Note that the adaptation rate is negative; however, in this paper we present adaptation rates as positive values to avoid confusion.

Every subject demonstrated trial-by-trial adaptation for all three control interfaces (Figures 2, **4**). When a visual perturbation was applied in the negative direction, the subject typically reacted to the perceived error by overcorrecting on the next trial. Thus, the slope of the regression line (solid line in Figure 2) reflects the degree to which the subject adapted to perturbations, and will be referred to here as the adaptation rate. Note that although the slope is always negative, here we present adaptation rates as positive values (correction opposite to perceived error) to avoid any confusion.

**Figure 3:**
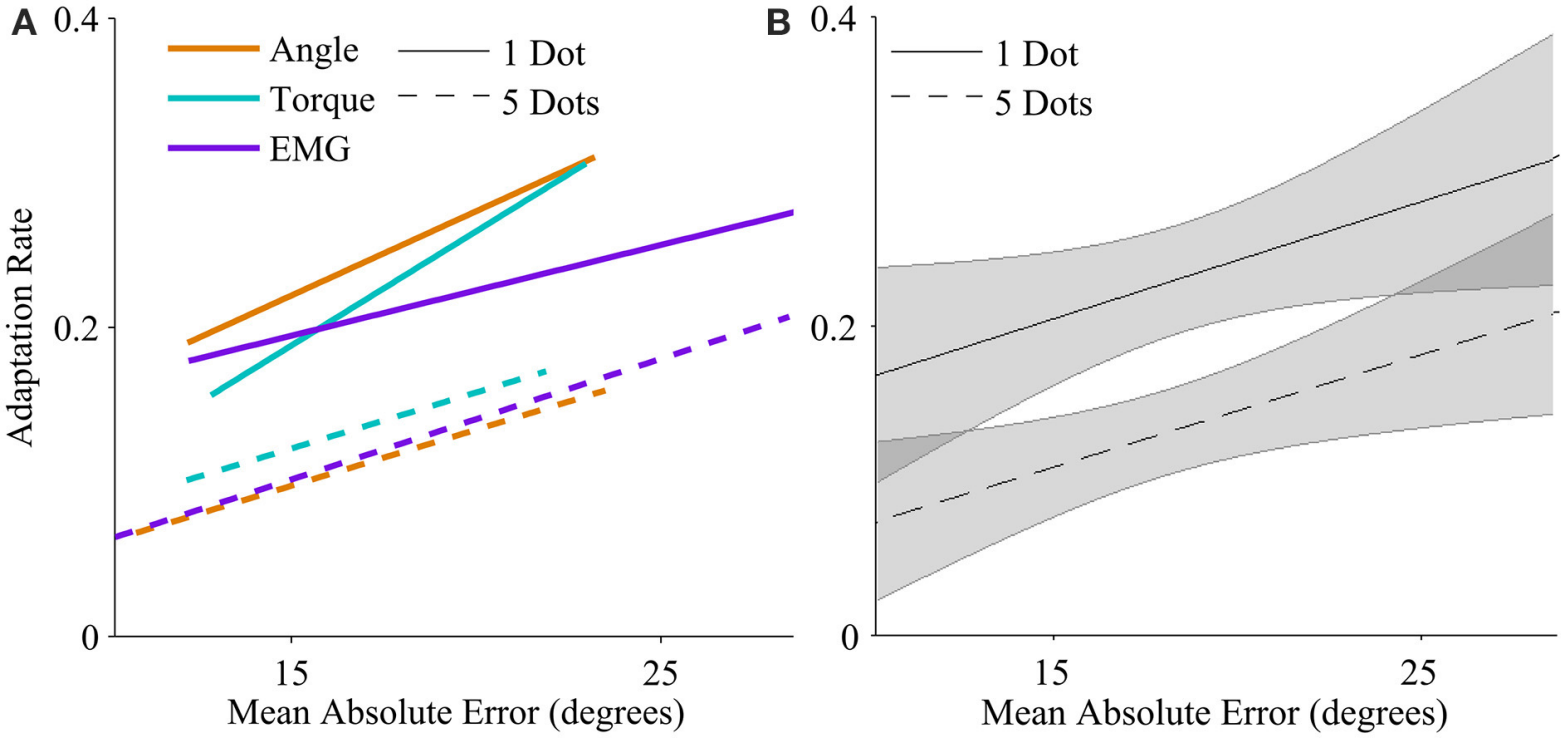
**Adaptation rate as a function of mean absolute endpoint error**. **(A)** Regression between adaptation rate and error with each control interface, for low feedback uncertainty (solid lines), and high feedback uncertainty (dashed lines). The range of each regression line runs from the minimum mean error to the maximum mean error across subjects. **(B)** Regression between adaptation rate and error across control interfaces for low feedback uncertainty (solid line), and high feedback uncertainty (dashed line). Shaded areas represent 95% confidence intervals of regression.

Higher mean errors significantly increased adaptation rate. The slope of the overall linear relationship between adaptation rate and mean error is statistically significant (*p* < 0.01, Table 1) and accounts for a large proportion of variance in adaptation rate (η^2^_*p*_ = 0.21, Table 1). This relationship depends on control interface and feedback condition (Figure 3A).

**Table 1:** **Results of Three-Way ANOVA on adaptation rate**.

**Factor**	**Type**	**Significance**	**Effect size**
Mean error	Continuous	*p* < 0.01	η^2^_*p*_ = 0.22
Control interface	Categorical	*p* = 0.89	η^2^_*p*_ = 0.01
Feedback uncertainty	Categorical	*p* < 0.01	η^2^_*p*_ = 0.32
(Control interface) × (Mean error)	Continuous	*p* = 0.78	η^2^_*p*_ = 0.01

Categorical factors test for an offset change in the dependent variable and continuous factors test for a slope change in the dependent variable. Effect sizes were assessed using partial eta squared, η^2^_p_ (Hentschke and Stüttgen, 2011; Richardson, 2011).

The control interface did not affect adaptation; there were no significant differences in adaptation rate between control interfaces (*p* = 0.7, η^2^_*p*_ = 0.01, Table 1). However, control interface did influence mean error. When using EMG control, subjects' mean errors were significantly higher than when using joint angle or torque control (Figure 5, *p* < 0.01, One-Way ANOVA with Tukey *post-hoc* tests).

Feedback uncertainty significantly affected adaptation rate for all three control interfaces (*p* < 0.01, η^2^_*p*_ = 0.32, Table 1). Higher feedback uncertainty decreased the intercept of the adaptation rate curve (Figure 3A). This means that subjects adapted less after trials with high feedback uncertainty, i.e., when terminal feedback was presented as five dots instead of one.

Various factors influenced adaptation rate (Figure 3B). Because control interface did not have a significant effect on adaptation rate, linear regressions were calculated and plotted across all three control interfaces. Mean error affected the slope of the adaptation curve and feedback uncertainty affected the intercept.

## Discussion

In this work we investigated how prosthesis control affects trial-by-trial adaptation by comparing three different control interfaces: joint angle, torque, and EMG. We found that the control interface did not significantly affect adaptation; instead adaptation rates depended primarily on mean error and on feedback uncertainty.

**Figure 4:**
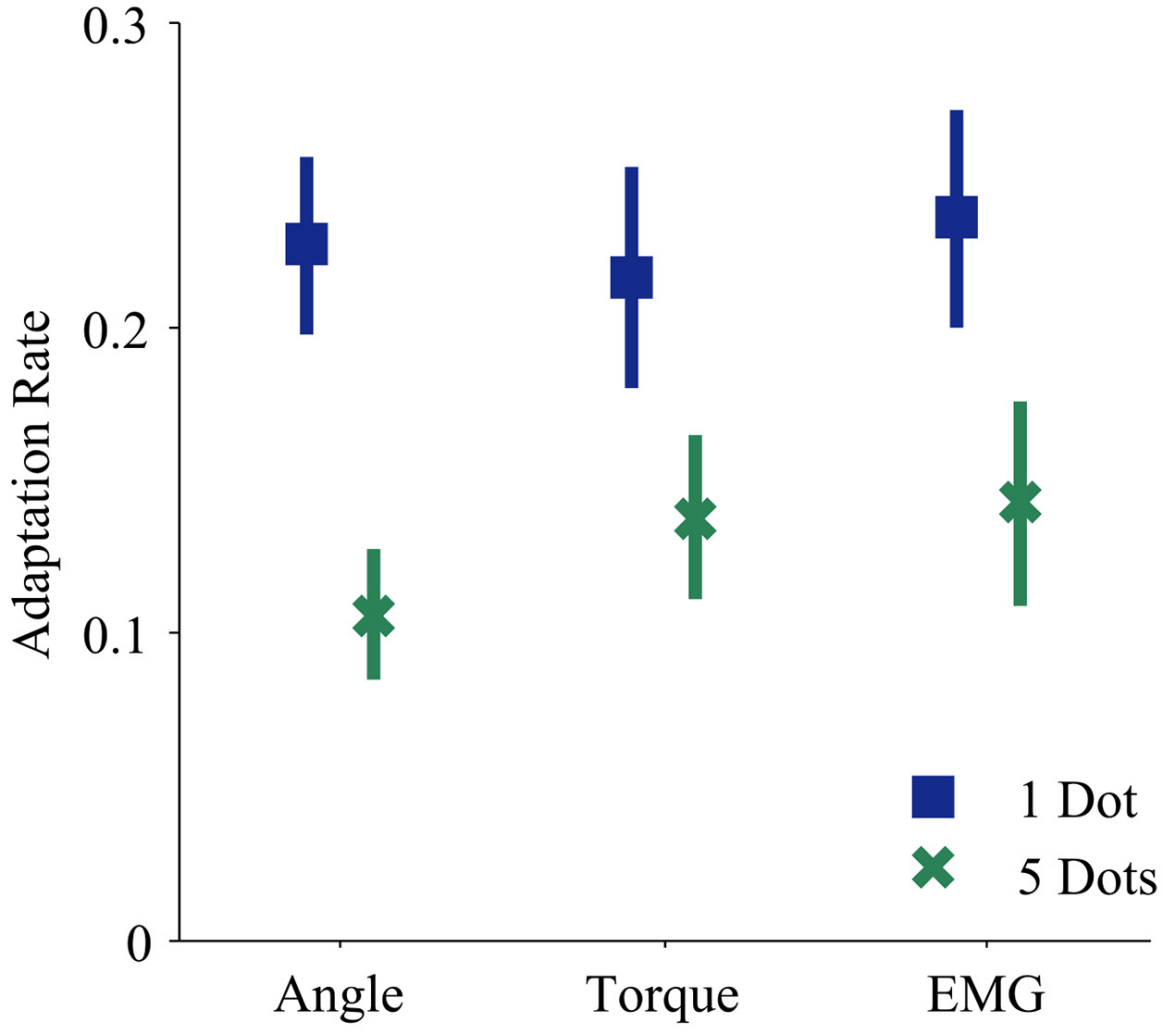
**Adaptation rates during each control modality and feedback condition**. Blue squares and green crosses represent the mean adaptation rates across subjects for one dot and five dot feedback, respectively. Bars represent standard errors of the mean.

Subjects were able to develop and adapt a simple internal model using EMG control (Figure 4). Previous studies show that amputees maintain the central nervous system capabilities needed for adaptation (Lotze et al., 1999, 2001). Other studies show that subjects adapt to novel transformations when using EMG control (Radhakrishnan et al., 2008). Our results support these findings and motivate future studies of adaptation behavior that requires more complex internal models during powered prosthesis control.

**Figure 5:**
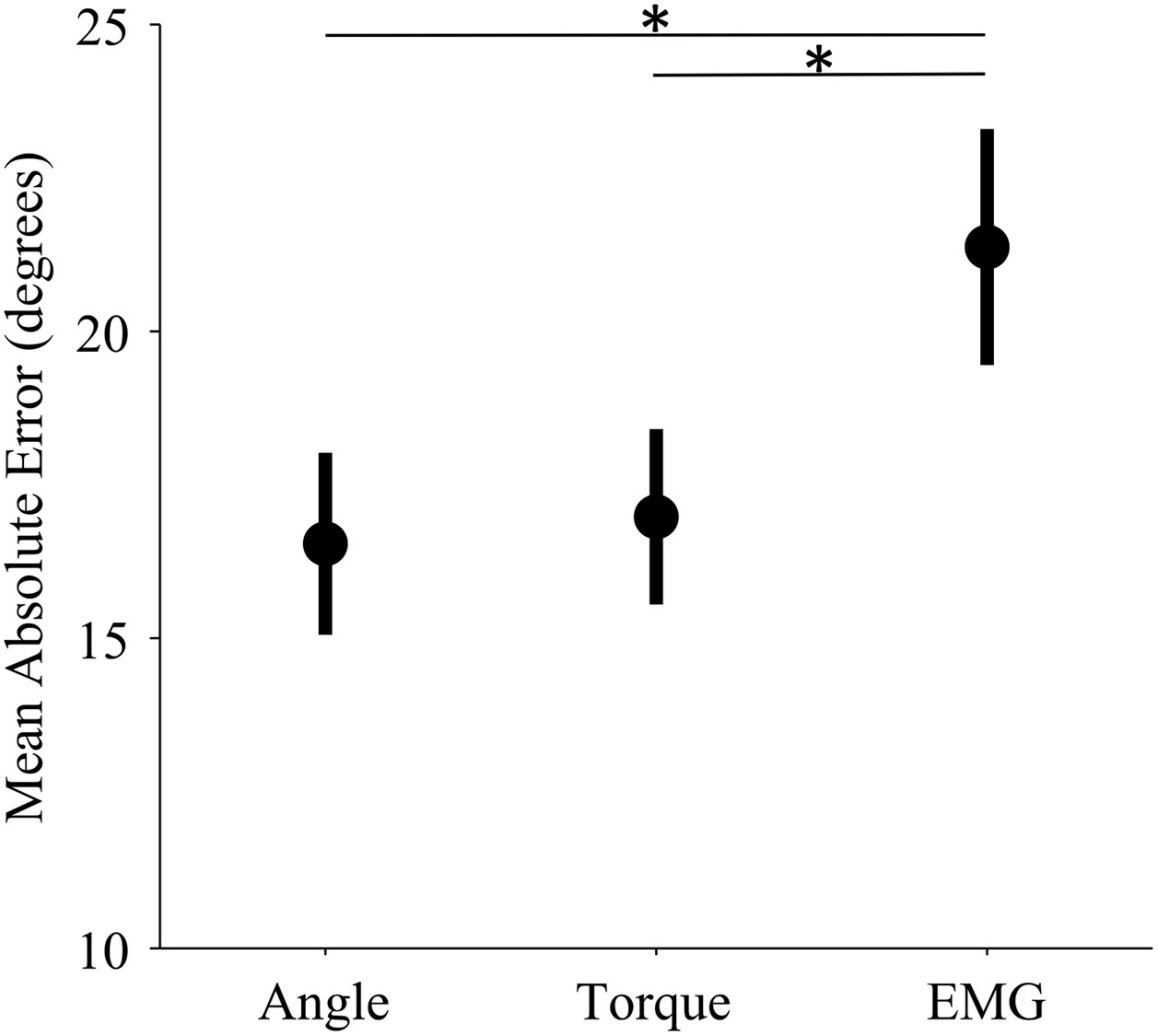
**Mean error levels for each control interface**. Markers represent the mean error across subjects for each control modality. Bars represent standard errors of the mean. Mean error refers to the mean absolute unperturbed endpoint error of all trials in a single experiment. (*) indicates significant difference (*p* < 0.01) as determined by a One-Way ANOVA with Tukey *post-hoc* multiple comparisons.

The relationship between mean error, feedback uncertainty, and adaptation rate supports the Bayesian framework, if we assume that mean error influences feedforward uncertainty. Bayesian theory predicts that feedforward uncertainty speeds adaptation and feedback uncertainty slows adaptation (Wei and Kording, 2010). This interaction of feedforward and feedback uncertainty is critical for the high uncertainty levels associated with powered prosthesis control. When viewed in light of this interaction, results of sensory feedback studies begin to form cohesive patterns. Sensory feedback reduces errors if feedforward control is noisy (Saunders and Vijayakumar, 2011) or if vision is removed (Wheeler et al., 2010), but has no significant effect in many other cases (Antfolk et al., 2013).

The patterns observed here have important implications for prosthesis control. When control is more precise, prosthesis users will rely less on feedback and more on their internal feedforward predictions. When sensory feedback is provided, the perceived uncertainty of this feedback determines whether there is any impact on control. Visual feedback also introduces another factor: if the uncertainty of sensory feedback is greater than that of visual feedback, it will not notably improve control over vision alone, since the two senses are integrated according to their uncertainty (Ernst and Banks, 2002).

The mean error of EMG control was significantly higher than that of both angle and torque control; however, adaptation rates of EMG control were not significantly different. The high mean error of EMG control is not surprising because EMG signals have higher variability than angle and torque signals (Vodovnik and Rebersek, 1974; Clancy et al., 2002). We offer two hypotheses for why we did not find a corresponding difference in adaptation rates. First, there may be a ceiling for adaptation rates. If subjects continually see very large errors, they may be so unsure of their feedforward signals that instead of adapting quickly, they do not adapt at all (e.g., Torres-Oviedo and Bastian, 2012). Second, increasing adaptation rate may not be optimal behavior in every situation. In this trial-by-trial adaptation paradigm, increasing adaptation means continually making large corrections in response to large errors. Furthermore, EMG noise is dependent on signal size; larger control signals (from stronger contractions) are more variable. Studies show that subjects learn to use smaller control signals in the presence of such signal-dependent noise (Chhabra and Jacobs, 2006). The noise characteristics of EMG control signals may have altered optimal adaptation behavior.

Higher mean error increased adaptation rates, and higher feedback uncertainty decreased adaptation rates, but control interface did not have a significant effect (Table 1 and Figure 3). Subjects behaved similarly when using different control modalities, including EMG signals. This result is encouraging, because it suggests that improved prosthesis control systems with lower errors may enable skilled, coordinated movement.

This study introduces the application of adaptation paradigms to powered prosthesis control; however, many questions remain. We chose a single DOF task for a simple initial comparison of EMG-controlled and able-bodied adaptation, but multi-DOF tasks might reveal differences and should be investigated. Similarly, only one muscle, the triceps brachii, was used for single-site proportional EMG control, whereas many powered prostheses are controlled by pattern recognition of multiple EMG signal features (Kuiken et al., 2009) or other multi-site control schemes (Zecca et al., 2002). Other limitations include the difficulties of selecting and matching control ranges for performance comparisons. Furthermore, this study included only able-bodied subjects interacting with a virtual environment. For amputees using physical prostheses, everyday tasks may involve higher levels of uncertainty from a greater variety of sources.

Our results provide a strong motivation for further investigation of adaptation behavior during powered prosthesis control. We found that subjects using EMG control adapted to perturbations in a manner consistent with Bayesian predictions. A better understanding of internal model development and adaptation will guide control and sensory feedback strategies to reduce uncertainty for prosthesis users.

### Conflict of interest statement

The reviewer Dr. Panagiotis Artemiadis declares that, despite having collaborated with Dr. Levi J. Hargrove on a review paper, the review process was handled objectively. The authors declare that the research was conducted in the absence of any commercial or financial relationships that could be construed as a potential conflict of interest.

## References

[B1] Antfolk C. D'Alonzo M. Rosén B. Lundborg G. Sebelius F. Cipriani C. (2013). Sensory feedback in upper limb prosthetics. Expert Rev. Med. Devices 10, 45–54 10.1586/erd.12.68 23278223

[B2] Baddeley R. J. Ingram H. A. Miall R. C. (2003). System identification applied to a visuomotor task: near-optimal human performance in a noisy changing task. J. Neurosci. 23, 3066–3075 12684493 10.1523/JNEUROSCI.23-07-03066.2003 PMC6742112

[B3] Biddiss E. A. Beaton D. Chau T. (2007). Consumer design priorities for upper limb prosthetics. Disabil. Rehabil. Assist. Technol. 2, 346–357 10.1080/17483100701714733 19263565

[B4] Biddiss E. A. Chau T. (2007). Upper limb prosthesis use and abandonment: a survey of the last 25 years. Prosthet. Orthot. Int. 31, 236–257 10.1080/03093640600994581 17979010

[B5] Burge J. Ernst M. O. Banks M. S. (2008). The statistical determinants of adaptation rate in human reaching. J. Vis. 8, 1–19 10.1167/8.4.20 18484859 PMC2684526

[B6] Chhabra M. Jacobs R. A. (2006). Near-optimal human adaptive control across different noise environments. J. Neurosci. 26, 10883–10887 10.1523/JNEUROSCI.2238-06.2006 17050726 PMC6674745

[B7] Clancy E. A. Morin E. Merletti R. (2002). Sampling, noise-reduction and amplitude estimation issues in surface electromyography. J. Electromyogr. Kinesiol. 12, 1–16 10.1016/S1050-6411(01)00033-5 11804807

[B8] Ernst M. O. Banks M. S. (2002). Humans integrate visual and haptic information in a statistically optimal fashion. Nature 415, 429–433 10.1038/415429a 11807554

[B9] Heckathorne C. (2004). Components for electric-powered systems, in Atlas of Amputations and Limb Deficiencies: Surgical, Prosthetic, and Rehabilitation Principles, eds Michael J. W. Bowker J. H. (Rosemont, IL: American Academy of Orthopaedic Surgeons), 145–172

[B10] Hentschke H. Stüttgen M. C. (2011). Computation of measures of effect size for neuroscience data sets. Eur. J. Neurosci. 34, 1887–1894 10.1111/j.1460-9568.2011.07902.x 22082031

[B11] Kawato M. (1999). Internal models for motor control and trajectory planning. Curr. Opin. Neurobiol. 9, 718–727 10.1016/S0959-4388(99)00028-8 10607637

[B12] Kording K. Wolpert D. M. (2004). Bayesian integration in sensorimotor learning. Nature 427, 244–247 10.1038/nature02169 14724638

[B13] Kuiken T. Li G. Lock B. Lipschutz R. D. Miller L. A. Stubblefield K. (2009). Targeted muscle reinnervation for real-time myoelectric control of multifunction artificial arms. J. Am. Med. Assoc. 301, 619–628 10.1001/jama.2009.116 19211469 PMC3036162

[B14] Lotze M. Flor H. Grodd W. Larbig W. Birbaumer N. (2001). Phantom movements and pain. An fMRI study in upper limb amputees. Brain 124, 2268–2277 10.1093/brain/124.11.2268 11673327

[B15] Lotze M. Grodd W. Birbaumer N. Erb M. Huse E. Flor H. (1999). Does use of a myoelectric prosthesis prevent cortical reorganization and phantom limb pain? Nat. Neurosci. 2, 501–502 10.1038/9145 10448212

[B16] Orbán G. Wolpert D. M. (2011). Representations of uncertainty in sensorimotor control. Curr. Opin. Neurobiol. 21, 629–635 10.1016/j.conb.2011.05.026 21689923

[B17] Parker P. A. Englehart K. Hudgins B. S. (2006). Myoelectric signal processing for control of powered limb prostheses. J. Electromyogr. Kinesiol. 16, 541–548 10.1016/j.jelekin.2006.08.006 17045489

[B18] Poulet J. F. Hedwig B. (2007). New insights into corollary discharges mediated by identified neural pathways. Trends Neurosci. 30, 14–21 10.1016/j.tins.2006.11.005 17137642

[B19] Radhakrishnan S. M. Baker S. N. Jackson A. (2008). Learning a novel myoelectric-controlled interface task. J. Neurophysiol. 100, 2397–2408 10.1152/jn.90614.2008 18667540 PMC2576223

[B20] Richardson J. T. E. (2011). Eta squared and partial eta squared as measures of effect size in educational research. Educ. Res. Rev. 6, 135–147 10.1016/j.edurev.2010.12.00114664681

[B21] Saunders I. Vijayakumar S. (2011). The role of feed-forward and feedback processes for closed-loop prosthesis control. J. Neuroeng. Rehabil. 8:60 10.1186/1743-0003-8-60 22032545 PMC3227590

[B22] Sensinger J. W. Weir R. F. (2008). User-modulated impedance control of a prosthetic elbow in unconstrained, perturbed motion. IEEE Trans. Biomed. Eng. 55, 1043–1055 10.1109/TBME.2007.905385 18334396 PMC10976976

[B23] Tassinari H. Hudson T. E. Landy M. S. (2006). Combining priors and noisy visual cues in a rapid pointing task. J. Neurosci. 26, 10154–10163 10.1523/JNEUROSCI.2779-06.2006 17021171 PMC6674625

[B24] Torres-Oviedo G. Bastian A. J. (2012). Natural error patterns enable transfer of motor learning to novel contexts. J. Neurophysiol. 107, 346–356 10.1152/jn.00570.2011 21957223 PMC3349698

[B25] Vodovnik L. Rebersek S. (1974). Information content of myo-control signals for orthotic and prosthetic systems. Arch. Phys. Med. Rehabil. 55, 52–56 4272626

[B26] Wei K. Kording K. (2009). Relevance of error: what drives motor adaptation? J. Neurophysiol. 101, 655–664 10.1152/jn.90545.2008 19019979 PMC2657056

[B27] Wei K. Kording K. (2010). Uncertainty of feedback and state estimation determines the speed of motor adaptation. Front. Comput. Neurosci. 4:11 10.3389/fncom.2010.00011 20485466 PMC2871692

[B28] Weir R. F. Sensinger J. W. (2009). The design of artificial arms and hands for prosthetic applications, in Biomedical Engineering and Design Handbook, Vol. 2, ed Kutz M. (New York, NY: McGraw-Hill), 537–598

[B29] Wheeler J. W. Bark K. Savall J. Cutkosky M. (2010). Investigation of rotational skin stretch for proprioceptive feedback with application to myoelectric systems. IEEE Trans. Neural Syst. Rehabil. Eng. 18, 58–66 10.1109/TNSRE.2009.2039602 20071271

[B30] Wolpert D. M. Ghahramani Z. Jordan M. I. (1995). An internal model for sensorimotor integration. Science 269, 1880–1882 10.1126/science.7569931 7569931

[B31] Zecca M. Micera S. Carrozza M. C. Dario P. (2002). Control of multifunctional prosthetic hands by processing the electromyographic signal. Crit. Rev. Biomed. Eng. 30, 459–485 10.1615/CritRevBiomedEng.v30.i456.80 12739757

